# Developmental Neuroendocrinology of Early-Life Stress: Impact on Child Development and Behavior

**DOI:** 10.2174/1570159X21666230810162344

**Published:** 2023-08-15

**Authors:** Nicolas C. Nicolaides, Christina Kanaka-Gantenbein, Panagiota Pervanidou

**Affiliations:** 1 Division of Endocrinology, Metabolism and Diabetes, First Department of Pediatrics, National and Kapodistrian University of Athens, School of Medicine, ‘Aghia Sophia’ Children's Hospital, Athens, 11527, Greece;; 2 Division of Endocrinology and Metabolism, Center of Clinical, Experimental Surgery and Translational Research, Biomedical Research Foundation of the Academy of Athens, Athens, 11527, Greece;; 3 School of Medicine, University Research Institute of Maternal and Child Health and Precision Medicine, National and Kapodistrian University of Athens, Athens, Greece;; 4 Department of Molecular Genetics, Function and Therapy, The Cyprus Institute of Neurology and Genetics, Nicosia, Cyprus;; 5 Unit of Developmental and Behavioral Pediatrics, First Department of Pediatrics, School of Medicine, National and Kapodistrian University of Athens, “Aghia Sophia” Children's Hospital, Athens, Greece

**Keywords:** Catecholamines, early-life stress, epigenetics, glucocorticoids, glucocorticoid receptor, neurodevelopmental disorders, stress system, stressors

## Abstract

Our internal balance, or homeostasis, is threatened or perceived as threatened by stressful stimuli, the stressors. The stress system is a highly conserved system that adjusts homeostasis to the resting state. Through the concurrent activation of the hypothalamic-pituitary-adrenal axis and the locus coeruleus/norepinephrine-autonomic nervous systems, the stress system provides the appropriate physical and behavioral responses, collectively termed as “stress response”, to restore homeostasis. If the stress response is prolonged, excessive or even inadequate, several acute or chronic stress-related pathologic conditions may develop in childhood, adolescence and adult life. On the other hand, early-life exposure to stressors has been recognized as a major contributing factor underlying the pathogenesis of non-communicable disorders, including neurodevelopmental disorders. Accumulating evidence suggests that early-life stress has been associated with an increased risk for attention deficit hyperactivity disorder and autism spectrum disorder in the offspring, although findings are still controversial. Nevertheless, at the molecular level, early-life stressors alter the chemical structure of cytosines located in the regulatory regions of genes, mostly through the addition of methyl groups. These epigenetic modifications result in the suppression of gene expression without changing the DNA sequence. In addition to DNA methylation, several lines of evidence support the role of non-coding RNAs in the evolving field of epigenetics. In this review article, we present the anatomical and functional components of the stress system, discuss the proper, in terms of quality and quantity, stress response, and provide an update on the impact of early-life stress on child development and behavior.

## 1 INTRODUCTION

The internal balance of all organisms, termed homeostasis, is constantly challenged by a large number of extrinsic or intrinsic stimuli, the stressors [1, 2]. Stress is defined as a state in which homeostasis is threatened or perceived as threatened by stressors [3]. To cope with stressful stimuli, organisms have a highly conserved neuroendocrine system, the stress system, which provides the appropriate physiological and behavioral responses to achieve and restore homeostasis [1-3]. If the stress response is inadequate, short-term,excessive or prolonged, it may have detrimental effects on fundamental physiologic functions, including growth, development, metabolism, reproduction, and the inflammatory or immune response [3].

The stress system consists of the hypothalamic-pituitary-adrenal (HPA) axis and the locus coeruleus/norepinephrine-autonomic nervous systems [1-5]. Glucocorticoids represent the end-products of the HPA axis and exert their numerous genomic and nongenomic actions through a ubiquitously expressed transcription factor, the glucocorticoid receptor (GR), which influences the transcription rate of an ever-increasing list of target genes [6-8]. On the other hand, catecholamines (adrenaline and noradrenaline) are secreted upon the concurrent activation of locus coeruleus/norepinephrine-autonomic nervous systems. Catecholamines act through binding on their cognate G Protein-Coupled Receptors (GPCRs), triggering rapid signal transduction pathways, thereby providing the “fight or flight or freeze” response upon exposure to stressors [9]. In this review, we present the anatomical components of the stress system, describe the repertoire of physical and behavioral alterations that occur during the stress response, and discuss the impact of exposure to early-life stressors on child development and behavior.

## 2 MATERIALS AND METHODS

A scientific literature search was conducted according to PRISMA guidelines at the PubMed database following a combination of search terms: (stress OR “stress system” OR “stress response” OR “early-life stress” OR neuroendocrinology) AND (neurodevelopment OR “neurodevelopmental disorders” OR “attention deficit hyperactivity disorder” OR “autism spectrum disorder” OR “post-traumatic stress disorder” OR “mental health” OR epigenetics OR “epigenetic modifications” OR COVID-19 OR “COVID-19 pandemic”).

## 3 THE STRESS SYSTEM

The stress system is formed by central and peripheral components. The central components are (i) the parvocellular neurons that release corticotropin-releasing hormone (CRH); (ii) the paraventricular nuclei (PVN) neurons of the hypothalamus that produce and secrete arginine vasopressin (AVP); iii) the CRH neurons of the paragigantocellular and parabrachial nuclei of the medulla and the locus coeruleus (LC); and (iv) other groups of neurons in the medulla and pons (LC/NE system), mostly releasing norepinephrine (NE). The central and peripheral components are (i) the HPA axis; (ii) the sympathetic-adrenomedullary systems (SNS); and (iii) components that are controlled by the parasympathetic system [10].

**Fig. (1):**
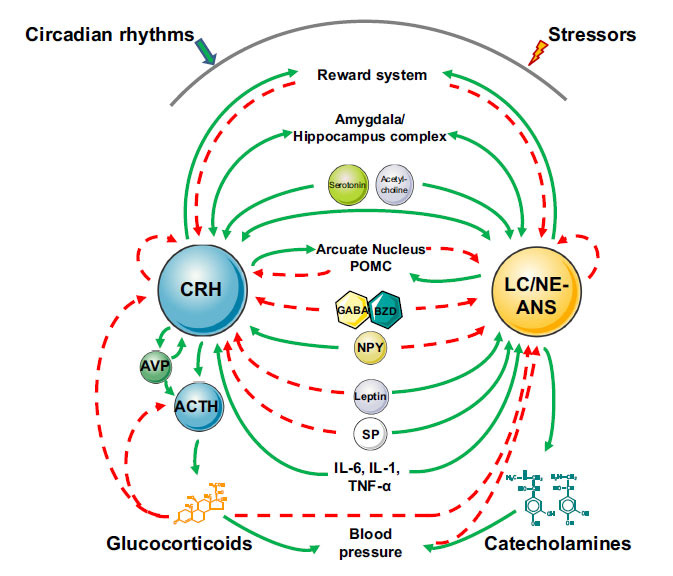
Interactions of the stress system with other homeostatic systems and molecules. Activation is represented by solid green lines, and inhibition by dashed red lines. **Abbreviations:** CRH: corticotropin-releasing hormone; ACTH: adrenocorticotropic hormone; AVP: arginine-vasopressin; LC/NE Symp Syst: locus coeruleus-norepinephrine/sympathetic nervous system; POMC: proopiomelanocortin; GABA: γ-aminobutyric acid; BZD: benzodiazepine; NPY: neuropeptide Y; SP: substance P. Modified from reference [2].

The components of the stress system interact with each other, as well as with other important loci/systems at multiple levels [1-5, 10, 11] (Fig. **1**). Indeed, CRH neurons cross-talk with the LC/NE system and *vice versa*. Moreover, the stress system activates the dopaminergic reward system, while it receives an inhibitory signal from the latter [1-5, 10, 11]. The stress system also sends stimulatory input to the central nucleus of the amygdala, which is responsible for the generation of fear and/or anger. Similarly, the central nucleus of the amygdala activates the stress system, closing the positive regulatory feedback loop [1-5, 10, 11]. The PVN of the hypothalamus, through CRH neurons, triggers the proopiomelanocortin (POMC)-containing neurons in the arcuate nucleus to secrete α-MSH and β-endorphin, which both have an inhibitory effect on the CRH and LC/NE systems. Furthermore, the HPA axis and the LC/NE systems respond to several neurochemical stimuli that stimulate (*e.g*., serotonin and acetylcholine) or inhibit [γ-aminobutyric acid (GABA) and benzodiazepines (BZD)] the activity of both components of the stress system [1-5, 10, 11]. Finally, leptin, the neuropeptide (NP) Y, and substance (S) P have been demonstrated to influence substantially the stress system activity [10, 11].

### 3.1 The HPA Axis

The HPA axis consists of the PVN of the hypothalamus, the anterior lobe of the pituitary gland and the adrenal cortex. Several extrinsic or intrinsic stressors trigger a group of neurons located in the PVN of the hypothalamus to produce and release CRH and AVP [12, 13]. CRH circulates through the hypophysial portal system and reaches the anterior lobe of the pituitary gland, where it binds to CRH receptors, leading to biosynthesis and secretion of ACTH into the peripheral systemic circulation [14, 15]. ACTH, then, binds to its transmembrane G-protein-coupled receptor of the adrenocortical cells located in the zona fasciculata of the adrenal cortex, activating the biosynthetic pathway of glucocorticoids (cortisol in humans, corticosterone in rodents) [16]. In addition, ACTH regulates the secretion of adrenal androgens and aldosterone by the zona reticularis and the zona glomerulosa, respectively.

Glucocorticoids are cholesterol-derived steroid hormones that contribute substantially to the maintenance of resting and stress-related homeostasis. These molecules regulate the cardiovascular tone properly, influence the intermediary metabolism through catabolic actions in the insulin-dependent organs, such as the liver, muscle and adipose tissue, and play a pivotal role in the inflammatory and immune response [17]. Glucocorticoids are also implicated in vital physiologic functions, such as growth, development, reproduction, behavior, and cognition, and are key factors in cell proliferation, differentiation and apoptosis (programmed cell death) [1-5, 18]. All these systemic glucocorticoid functions are mediated by a ubiquitously expressed intracellular receptor, the human glucocorticoid receptor (hGR), which is a member of the steroid receptor family of the nuclear receptor superfamily of transcription factors [6-8, 17, 18].

The *NR3C1* gene in humans is located on chromosome 5 and consists of 10 exons [19]. Exon 1 contains the 5’-untranslated region, whereas exons 2-9α and 9β encode the functional protein. The alternative use of 9α or 9β generates the two main protein isoforms, the hGRα and the hGRβ [6-8, 17, 18]. The hGRα is the classic GR that mediates all the above-discussed glucocorticoid actions. The hGRβ functions as an inhibitor of hGRα-mediated transcriptional activity and influences the transcription of several genes in a positive or negative fashion, independently of hGRα [19]. More than a decade ago, Lu and Cidlowski demonstrated that the translation process of hGRα mRNA might begin from eight alternative sites through leaky ribosomal scanning or ribosomal shunting, giving rise to eight different hGRα protein isoforms [20]. We speculate that the same translation mechanisms might occur to possibly generate eight distinct hGRβ proteins [18, 19].

**Fig. (2):**
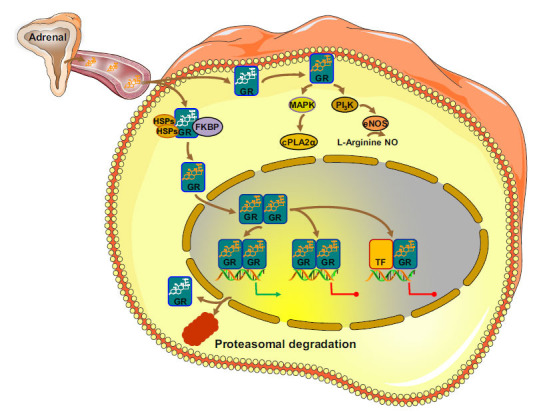
Genomic and nongenomic glucocorticoid signaling pathways. **Abbreviations:** GR: glucocorticoid receptor; HSP: heat shock proteins; FKBP: immunophilins; MAPK: mitogen-activated protein kinases; cPLA2α: cytosolic phospholipase A2 alpha; PI3K: phosphatidylinositol 3-kinase; eNOS: endothelial nitric oxide synthetase; NO: nitric oxide; TF: transcription factor.

Within the glucocorticoid-target cells, the unbound hGRα is primarily cytoplasmic, forming a multimember complex with other proteins, including the chaperon heat shock proteins (HSPs) 90 and 70 and immunophilins (Fig. **2**). Upon glucocorticoid-binding, hGRα dissociates from the protein partners, moves into the nucleus, and forms homo- or hetero-dimers that bind onto specific DNA sequences, the glucocorticoid response elements (GREs), within the regulatory regions of glucocorticoid-responsive genes, influencing the transcription rate of the latter in a positive or negative fashion [6-8, 17-19] (Fig. **2**). Alternatively, the ligand-activated hGRα might regulate gene expression independently of GRE-binding by physically interacting, as a monomer, with other well-studied transcription factors, including the nuclear factor-κB (NF-κB), the activator protein-1 (cFos/cJun, AP-1), p53 and members of signal transducers and activators of transcription (STATs), thus inducing or repressing their transcriptional activity [6-8, 17-19]. In addition, the transcription factor “circadian locomotor output cycle kaput” (CLOCK), which interacts with the brain–muscle–arnt-like protein 1 (BMAL1), has been identified as a novel partner of hGRα. CLOCK acetylates several lysine residues located within the hinge region of the receptor, thus leading to decreased binding onto GREs and reduced hGRα-mediated transcriptional activity [21]. Subsequent *in vivo* and *ex vivo* studies reported that the acetylation status of hGRα was higher in the morning and lower at night, preventing tissue exposure to excessive circulating concentrations of glucocorticoids [22].

Further to genomic actions, glucocorticoids can induce a growing number of systemic actions within a short time frame. These glucocorticoid actions, termed nongenomic, occur within seconds or minutes and do not require transcription or translation [23]. Examples of nongenomic glucocorticoid actions include (i) the immediate glucocorticoid-induced suppression of ACTH secretion from the anterior lobe of the pituitary [24]; (ii) the rapid increase in the frequency of postsynaptic excitatory potentials in the hippocampus by glucocorticoids [25]; (iii) the immediate reduction of blood pressure and the increase in blood flow of coronary and cerebral vessels in patients with myocardial infarction or stroke that receive synthetic glucocorticoids [26]; and (iv) the rapid disruption of T-cell receptor (TCR) signaling by glucocorticoids [27]. The nongenomic glucocorticoid actions are mediated by membrane-anchored GRs, which upon activation, trigger rapid kinase signaling pathways, including the mitogen-activated protein kinase (MAPK) or the phosphatidylinositol 3-kinase (PI3K) pathways [28].

### 3.2 The LC-NE, Systemic Sympathetic, Adrenomedullary and Parasympathetic Systems

The autonomic nervous system consists of the sympathetic and parasympathetic systems that regulate a large number of physiologic systems, including the respiratory, cardiovascular, gastrointestinal, neuroendocrine, and central nervous systems. The sympathetic system, *via* activation of the adrenal medulla, contributes substantially to the “fight or flight or freeze reaction” by releasing adrenaline and noradrenaline [2-4, 9, 11]. On the contrary, the parasympathetic system either antagonizes or even assists the functions of the sympathetic system by enhancing or decreasing its activity, respectively [2-4, 9, 11]. The sympathetic and parasympathetic systems employ acetylcholine and norepinephrine, several neuropeptides and a growing list of lipid mediators of inflammation, adenosine triphosphate (ATP) or nitric oxide in order to transduce the neural signal [2-4, 9, 11].

## 4 THE STRESS RESPONSE

Upon exposure to stressors, the stress system provides the adaptive stress response *via* two serial waves of hormonal secretion. The first wave occurs within seconds and is characterized by the increased secretion of catecholamines from the sympathetic nervous system, followed by elevated CRH release from the hypothalamus into the hypophysial portal vein system and increased release of ACTH from the anterior lobe of the pituitary. Subsequently, GnRH levels decrease, leading to reduced secretion of FSH and LH. Finally, prolactin (PRL) and growth hormone (GH) levels increase with concurrently increased secretion of glucagon from the pancreas [29]. The second wave of hormonal secretion is characterized by a slower tempo and involves glucocorticoids [29]. All these stress-induced hormonal alterations during the two serial waves are translated in cellular effects; therefore, the hormones that participate in the first wave trigger rapid signal transduction pathways through GPCRs and second messengers, whereas glucocorticoids bind onto the GR, which influences the transcription of 20% of human expressed genes, thereby requiring longer time from at least 20 min to hours to complete the stress response [30].

Accumulating evidence suggests that some of the nongenomic glucocorticoid actions undoubtedly play a fundamental role in the acute phase of the stress response [31, 32]. In addition to activating membrane GRs, glucocorticoids trigger the mineralocorticoid receptor (MR) even at lower concentrations than those activating the GR. Upon glucocorticoid binding to the membrane or cytoplasmic MRs, the activity of multiple kinase signaling pathways increases. Importantly, the membrane GRs inhibit glutamatergic neurotransmission, whereas the membrane MRs facilitate glutamatergic neurotransmission. At post-synaptic neurons, glucocorticoids activate membrane-localized GRs that trigger the c-AMP-PKA pathway and increase the production and secretion of anandamide (AEA) and 2-arachidonoylglycerol (2-AG). Both these molecules activate the cannabinoid receptors type 1 that inhibit the secretion of glutamate-containing vesicles at the pre-synaptic level [31-33]. In contradistinction, both pre- and post-synaptic membrane MRs facilitate glutamatergic transmission. Indeed, presynaptically, glucocorticoids bind at low concentrations to membrane MRs that activate the extracellular signal-regulated kinase (ERK) cascade, facilitating glutamatergic neurotransmission. At the same time, the postsynaptic glucocorticoid-induced activation of membrane MRs inhibits the activity of potassium IA-currents, thereby facilitating the diffusion of membrane AMPA receptors [31, 32].

Through the above-described neuroendocrine mechanisms, several behavioral and physical adaptation alterations occur, increasing the chances of survival [1-4, 9-11]. Indeed, during acute stress, organisms often present with increased arousal, alertness, improved cognition, vigilance, focused attention, as well as euphoria, enhanced pain tolerance, decreased feeding and inhibition of reproductive function. Moreover, physical adaptation changes take place upon exposure to stressors. Oxygen and nutrients are supplied to the CNS, and the stressed organism employs a higher respiratory rate, an increased cardiovascular tone and a shift of intermediary metabolism towards catabolism in order to respond properly to stressful stimuli [34]. Furthermore, organisms activate counteracting mechanisms to prevent an over-response from the stress system [1-4, 9-11]. If these mechanisms do not succeed in controlling the quantity and the quality of stress-related adaptive changes, the stress response may become excessive, prolonged and maladaptive, leading to the development of several acute or chronic stress-related pathologic conditions.

## 5 THE IMPACT OF PRENATAL MATERNAL STRESS ON NEURODEVELOPMENT

The intrauterine life has been undoubtedly recognized as a critical developmental period during which stress mediators might be transmitted from mother to child. Indeed, fetal development could be affected by several maternal, placental and fetal factors, leading to neurodevelopmental and/or behavioral alterations at different time points during pregnancy, in a process termed “fetal programming” [35].

Exposure to glucocorticoids could potentially lead to structural or functional alterations of the fetal central nervous system. In animal models, fetal exposure to increased circulating concentrations of cortisol may have a detrimental impact on the neurogenesis of the hippocampus [36]. In humans, *in-utero* exposure to synthetic glucocorticoids led to a thinner cortex in the offspring [37]. In addition, the elevated levels of glucocorticoids may increase the placental CRH, which is considered a significant predictor of preterm birth and intrauterine growth restriction, leading to potential alterations of the activity of fetal brain CRH receptors [36].

The placenta plays a crucial role in controlling fetal exposure to maternal cortisol by expressing the enzyme 11β-hydroxysteroid dehydrogenase type 2 (11b-HSD2). This enzyme catalyzes the conversion of active cortisol to inactive cortisone; therefore, 11b-HSD2 prevents the transfer of elevated glucocorticoids from mother to fetus. Any prenatal maternal stressor might alter the expression and/or the activity of 11b-HSD2, thereby leaving the fetus unprotected from the deleterious effects of the abnormally elevated glucocorticoid levels [36]. These increased glucocorticoid concentrations have been shown to influence the production and metabolism of other placental products, such as prostaglandins, lactogen, progesterone, as well as glucose transporters [36]. In addition to glucocorticoids, prenatal maternal stress also increases the production of catecholamines that affect blood flow and, thus, placental function. Moreover, the placental oxygenation status seems to contribute to placental homeostasis since placental hypoxia has been associated with the excessive production of inflammatory cytokines, including interleukin-6 (IL-6), interleukin 1β (IL-1β), and tumor necrosis factor-a (TNF-α). Other studies have demonstrated that maternal stress and placental hypoxia alter the ability of neurons to migrate and undergo myelination [38].

A growing body of evidence suggests that prenatal maternal stress may cause alterations in the offspring’s HPA axis. Indeed, maternal stressors during pregnancy lead to increased basal glucocorticoid secretion and alterations in the stress response of offspring. The expression of GR in the hippocampus was defective following exposure to stressors, posing a negative impact on postnatal development [36]. Moreover, the activity of the hypothalamic-pituitary-thyroid (HPT) axis may be negatively affected by exposure to maternal stressors, thereby altering fetal brain development [39]. Maternal depression and maternal stress-related pathologic conditions associated with increased cortisol concentrations might reduce maternal circulating thyroid hormones, leading to decreased levels of maternal T4 that reach the fetal brain through the placenta [39]. Since thyroid hormones are *sine qua non* for neurogenesis and synaptogenesis of the fetal brain throughout pregnancy, the glucocorticoid-mediated suppression of the HPT axis could be another potential mechanism through which prenatal maternal stress impacts neurodevelopment in a negative fashion.

## 6 EARLY-LIFE STRESS (ELS) AND NEURODEVELOPMENTAL DISORDERS

Several lines of evidence have proved the high risk for attention deficit hyperactivity disorder (ADHD) and autism spectrum disorder (ASD) in the offspring following exposure to prenatal maternal stressors or/and postnatal environmental stressors [35]. Importantly, maternal cortisol concentrations and maternal psychosocial stress substantially influence cognitive functions at one year of age among healthy full-term infants, indicating the important role of these factors in fetal programming [40]. In addition, adverse childhood experiences (ACEs) have been associated with an increased risk for anxiety, depression, suicide attempts, behavioral problems, ADHD, and substance use disorder, both in males and females aged 12-17 years [41].

### 6.1 ELS and ADHD

Prenatal exposure to bereavement has been associated with a higher risk of ADHD in the offspring [42]. Similarly, the largest population-based study to date demonstrated that prenatal maternal stress during the third trimester of pregnancy (death of a first-degree relative) caused an increased risk for ADHD and ASD in the offspring, while stress due to bereavement during the second postnatal year led to high risk only for ASD [43]. Consequently, the developmental period affected may be important [35, 40]. Moreover, depressed mood and/or anhedonia during pregnancy are also associated with ADHD and ASD in the offspring [44].

The association between prenatal maternal stress and ADHD seems to be sex-specific, specifically stronger in boys compared to girls [45, 46]. The exposure to prenatal maternal stressors during the beginning of the pregnancy was found to be independently related to the symptoms of ADHD in children aged 7 years old, especially in boys, and independently of other sociodemographic factors or smoking [45]. Moreover, prenatal anxiety during at least two trimesters caused increased secretion of placental inflammatory cytokines, mostly in boys than in girls [46]. The severity of ADHD symptoms was associated with the severity of stressors since children aged 6-12 years with ADHD, whose mothers experienced moderate/severe stress during pregnancy, presented with more severe symptoms than children whose mothers were not exposed to prenatal stressors [47].

Several studies have shown the association between ACEs and the presence and severity of ADHD symptoms in children or adolescents. Brown and collaborators found that children aged 4-17 years with ADHD had higher exposure to ACEs compared to controls [48]. Importantly, the ACE score was found to be associated with the severity of ADHD symptoms [48]. In another study, Walker *et al.* demonstrated a higher prevalence of ADHD upon cumulative exposure to ACEs [49]. These results indicated that combined exposure to ACEs has a stronger association with ADHD than the presence of a single ACE in childhood/adolescence [49, 50]. Furthermore, ACEs at age 5, including emotional/physical abuse or neglect, parental domestic violence, substance abuse, anxiety and/or depression, are related to externalizing/ internalizing behaviors and a possible diagnosis of ADHD at the age of 9 [51]. Moreover, ACEs, either before the age of 5 or between the age of 5 and 9 years, are associated with a diagnosis of ADHD at the age of 9 [52]. Finally, the type of ACE may also play an important role in the relationship between ELS and ADHD. Indeed, early maltreatment is strongly associated with both diagnosis and symptoms of ADHD in childhood and adolescence [53]. For girls, physical abuse was reported to increase the risk for ADHD diagnosis, whereas, in boys, the type of ELS that increased the likelihood of ADHD was emotional abuse [53].

### 6.2 ELS and ASD

Several maternal factors, such as a history of childhood abuse [54] or exposure to hurricanes, storms or other natural disasters [55], have been associated with increased prevalence of risk for ASD. Not only maternal factors but also several adverse perinatal conditions, including prior abortion, partner abuse, depression, alcohol use, smoking, gestational diabetes, preeclampsia, duration of pregnancy of less than 37 weeks and low birth weight, contribute to this increased risk [54]. Even before pregnancy, the risk for ASD was reported to be increased in children whose mothers experienced intimate partner violence in the 2 years before the child’s birth, independently of other confounding factors [56]. However, two large population-based prospective studies failed to support any association between exposure to ELS before or during pregnancy or even during three years postnatally with an increased risk for ASD [57].

The timing of exposure to ELS seems to be associated with ASD [58]. Class and collaborators found that the death of a first-degree relative during the third trimester of pregnancy or during the second postnatal year led to an increased risk for ASD [48]. Moreover, children whose mothers were exposed to prenatal stressors at 21-32 weeks, with a peak at 25-28 weeks of gestation, had a higher incidence of ASD [59].

Finally, children with ASD may be at increased risk for exposure to ACEs [60-64]. A study by Kerns *et al.* found that the association between ACEs and ASD may be influenced by family income and mental health conditions [61]. Moreover, children with ASD and a history of exposure to many ACEs are at increased risk for mental health problems, and the diagnosis and treatment initiation may be delayed [63].

Beyond the effects of prenatal and early postnatal life stress on child development and behavior [64], the stress system serves as a major adaptive physiological mechanism in humans. Several studies have shown an altered activity (hyper- or hypo-activation) of the stress system, as evidenced by altered concentrations of stress-related biomarkers and brain changes in individuals with neurodevelopmental disorders (*e.g*., Autism and Attention Deficit Hyperactivity Disorder (ADHD)) or traits related to altered neurodevelopment (*e.g*., deficits in cognition, executive functions, *etc*.). Children with neurodevelopmental disorders often exhibit non-typical functions of the HPA axis and the LC-NE/ANS, both at the resting state and during social and/or other environmental stressors [65-69]. In addition, children with neurodevelopmental disorders may appear with greater comorbidity of mental and physical conditions, possibly relevant to altered stress development [69].

## 7 ELS, POSTTRAUMATIC STRESS DISORDER, AND MENTAL HEALTH CONSEQUENCES

Further to the relationship between ELS and neurodevelopmental disorders that appear in the first years of life, ELS has been associated with several clinical conditions. There is abundant evidence from both retrospective and prospective studies revealing the negative impact of ELS on child and adult mental and physical health, although many who experienced childhood traumatic stress are resilient [70]. A higher risk of post-traumatic stress disorder (PTSD), depression, schizophrenia, suicide attempts and risk behavior patterns (*i.e*., tobacco and alcohol consumption) in later life have been highly associated with ELS experiences [70-74]. Exposure to traumatic events is rather common in childhood, and PTSD will occur in a significant portion of those experiencing adverse traumatic stressors [65]. Estimates of the incidence of PTSD in children vary by sample, methodologies, and different types of traumatic events [75]. Roughly two-thirds of youth are exposed to trauma by the age of 17 years, and approximately 5% meet lifetime criteria for PTSD [76, 77]. PTSD describes the clustering of symptoms that develop after exposure to traumatic life events involving actual or threatened death, serious injury, or sexual violence. PTSD, more recently, has been reconceptualized in order to include a broader spectrum of negative responses to traumatic experiences. The DSM-5 relocated the disorder to a new chapter comprising trauma and stressor-related disorders. In addition, a subtype for children younger than 6 years was created, lowering the pediatric diagnostic thresholds. The PTSD diagnosis includes symptoms of intrusion, avoidance, negative alterations in cognition and mood, and alterations in arousal and reactivity [78]. Until recently, the diagnostic criteria of PTSD are not developmentally appropriate, mainly in very young children. However, DSM-5 provides an extensive description of developmental differences in symptom expression and developmentally modified criteria for children aged 6 years and younger. This set of developmentally sensitive criteria includes three symptom groups: i. intrusion, ii. persistent avoidance/negative alterations in cognition and mood, and iii. alterations in arousal and reactivity [78].

Several studies have investigated the neuroendocrine alterations in adults with PTSD. The majority of studies have reported increased basal levels of corticotropin-releasing hormone (CRH) in cerebrospinal fluid (CSF) and decreased circadian cortisol levels in the periphery, either in the saliva, the serum or in urine [71, 72]. However, some studies, especially on younger individuals, have demonstrated divergent findings comprising increased peripheral cortisol concentrations in comparison to healthy individuals [79-81]. Regarding the functioning of the SNS, catecholamine levels in CSF, urine, and plasma have been consistently found to be increased in individuals with PTSD [79, 80, 82].

Children and adolescents may exhibit different neuroendocrine responses to acute or chronic stressors compared to adults [71-73]. In one of the first neurobiological studies on sexually abused girls with a history of depressive symptomatology, an elevation in 24-hour urine catecholamines concentrations was found compared to matched controls [83]. In another study, children with a history of maltreatment exhibited a significant variation in morning/afternoon cortisol concentrations, depending on the type of trauma. In this group, morning salivary cortisol concentrations were significantly elevated in children with a history of physical and sexual abuse and children who had been neglected or emotionally abused, whereas children who had experienced only physical abuse exhibited lower morning cortisol concentrations [84]. Regarding the SNS system, an asymmetry was revealed between salivary alpha-amylase (sAA) concentrations and cortisol responses to a social stressor in maltreated adolescents [85]. This asymmetry might be caused by interrupting the synchrony between the two components of the stress system or by causing attenuation in one of them but not the other [85]. Another study on maltreated adolescent girls revealed an attenuated response of the HPA axis in response to the Trier Social Stress Test (TSST), whereas the control group exhibited an increase in cortisol levels following the TSST, which gradually decreased over time [86]. Finally, a long-term longitudinal study that assessed the non-stress cortisol at six-time points, from age 6 to age 30, in girls with chronic trauma of familial maltreatment demonstrated that the linear trend for abused females was significantly less [87]. These findings, in chronically stressed young individuals, provided evidence that cortisol hyposecretion may follow a period of increased cortisol secretion, which is referred to as the “attenuation hypothesis” of cortisol secretion, observed in chronically traumatized individuals.

The effects of acute stressors, such as natural disasters or accidents, on child development and behavior are less studied. A study after the 1988 earthquake in Armenia on adolescents who lived at different distances from the epicenter of the earthquake demonstrated that the more symptomatic adolescents living in the city closer to the epicenter had lower mean baseline morning cortisol levels, greater day 2 cortisol suppression after dexamethasone, and a more rapid decline in 3-methoxy-4-hydroxyphenylglycol (MHPG) levels over the day 1 [88]. A study conducted by our group investigated the neuroendocrine profiles of children and adolescents aged 7-18 involved in motor vehicle accidents (MVAs) in relation to the development of PTSD diagnosis. Children were assessed immediately after hospitalization and 1 and 6 months later [89, 90]. The group that later developed and maintained PTSD diagnosis had higher serum interleukin-6 (IL-6) and evening salivary cortisol concentrations [89] compared to those that did not develop PTSD. In addition, a progressive elevation of plasma noradrenaline was noted in months 1 and 6, together with a gradual normalization of evening salivary cortisol concentrations in children with chronic PTSD compared to the non-PTSD and the control group [90]. This study revealed a divergence of cortisol and noradrenaline concentration over time in those who developed and maintained PTSD diagnosis.

The effects of ELS on the activity of the HPA and the SNS axes seem to depend on the developmental time window affected, the sex and age of the individual, previous exposure to stress and trauma, the type and chronicity of the stressor, the compensating mechanisms, as well as the environment [91].

## 8 ADVERSE CHILDHOOD EXPERIENCES (ACES) AND PSYCHOPATHOLOGY IN CHILDHOOD AND ADOLESCENCE

**Fig. (3):**
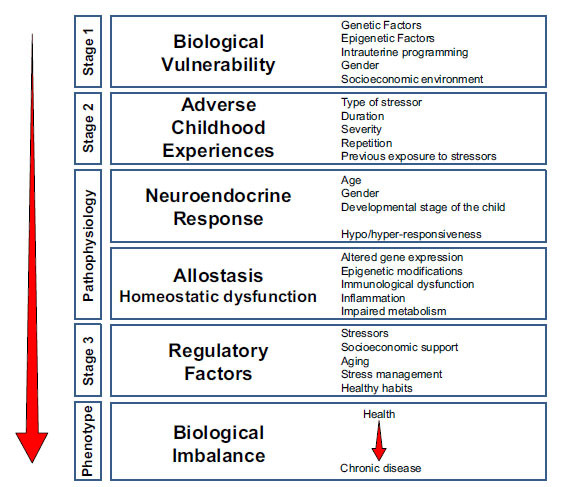
Neurobiological trajectories from adverse childhood experiences to chronic diseases. Modified from reference [95].

The term Adverse Childhood Experiences (ACEs) has also been used to describe a wide range of traumatic or stressful experiences during childhood and adolescence. Children and adolescents who experienced maltreatment and other Adverse Childhood Experiences (ACEs) are at increased risk for developing borderline personality disorder symptoms [92], major depressive disorder or bipolar disorder [93, 94]. Moreover, ACEs have been demonstrated as a predictor of worse outcomes during anti-depressant treatments, although a recent systematic review did not report a difference in treatment response among depressive patients with or without a history of childhood maltreatment [94]. However, several lines of evidence suggest that there are well-described neurobiological trajectories that link ACEs to chronic diseases, including several psychopathologies in childhood and adolescents [95, 96] (Fig. **3**). Several biological factors, such as genetic and epigenetic factors, sex, as well as the intrauterine and socioeconomic environment, represent a vulnerable substrate on which ACEs act as acute or chronic stressors of different severity and duration. If the stress response is inappropriate in terms of quantity or quality, homeostasis is turned into allostasis through altered gene expression, epigenetic modifications, immunological dysfunction, inflammation and impaired metabolism [95, 96] (Fig. **3**). All these factors contribute substantially to the development of contemporary non-communicable chronic disorders, including metabolic and/or neuropsychiatric diseases.

## 9 ELS IN THE EPIGENETIC ERA

“Epigenetics” is an Aristotelian term that refers to molecular events “above the genome”, leading to alterations in gene expression without changes in the DNA sequence [97]. Epigenetics is an evolving field of biology and medicine, which studies the dynamic interaction between genes and the environment. These interactions have been confirmed by several epidemiological studies [98]; however, the specific molecular mechanisms through which the social and physical environment turn gene expression “on” and “off” are currently under intense investigation. The tremendous progress of molecular biology in association with the well-recognized advances in biomedical technology has provided a deeper understanding of how the human expressed genome is reprogrammed upon exposure to an ever-increasing number of stressors, including ELS.

**Fig. (4):**
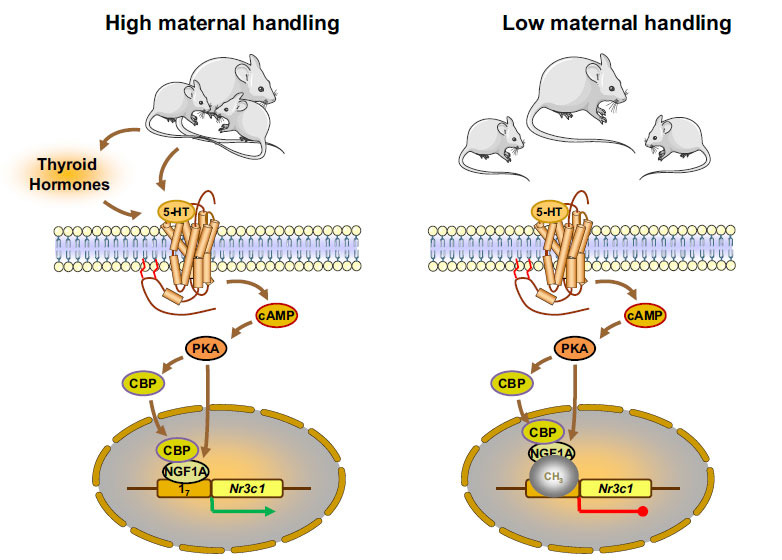
Signaling pathways linking maternal handling with hippocampal GR expression. **Abbreviations:** 5-HT: serotonin; cAMP: cyclic-AMP; PKA: protein kinase A; CBP: cyclic AMP response element-binding (CREB) protein; NGFIA: nerve growth factor-inducible A; Nr3c1: nuclear receptor subfamily 3, group C, member 1 (GR) gene. Modified from reference [3].

Almost four decades ago, a report by Michael J. Meaney and his collaborators provided for the first time evidence supporting the effect of ELS on the expression of the *NR3C1* gene that encodes GR [99]. The researchers compared a number of rats exposed to maternal licking/grooming with unhandled ones as an animal model of ELS and reported that handled rats had statistically significant increased hippocampal GRs compared to the unhandled rats [67]. Subsequent molecular and cellular studies by the same research team found that high maternal licking/grooming led to increased thyroid hormone concentrations, enhanced serotonin signaling, and increased binding of the transcription factor nerve growth factor inducible factor A (NGFI-A) to a specific region of exon 1_7_ of the *NR3C1* gene inducing the expression of hippocampal GR (Fig. **4**) [100-104]. On the other hand, low maternal licking/grooming resulted in increased methylation of cytosines within the exon 1_7_ of the *NR3C1* gene preventing the binding of NGFI-A onto exon 1_7_ and leading to decreased hippocampal GR expression (Fig. **4**) [105]. It was found that methylation of DNA is the most frequent and well-studied epigenetic modification that largely occurs at cytosine bases, followed by guanine bases (CpG islands) in the mammalian genome. The methylated cytosine inhibits the binding of transcription factors to regulatory regions of several genes, ultimately leading to the suppression of gene expression without any alterations in DNA sequence [106]. In addition to DNA methylation, accumulating evidence suggests that several types of non-coding RNAs (ncRNAs), including micro-RNAs (miRs) and long non-coding RNA (lncRNAs) participate in gene repression post-transcriptionally through targeting the mRNAs, thereby prompting the degradation of the latter and resulting in low protein expression [107, 108].

In addition to GR, many studies have shown that the list of genes implicated in stress response and undergoing epigenetic modifications is rapidly increasing. Among them, the CRH, glial-derived neurotrophic factor (GDNF), brain-derived neurotrophic factor (BDNF) and arginine-vasopressin (AVP) genes were thoroughly studied, either in different neurons or from heterogeneous brain tissues [107]. Although epigenetic modifications are inherited by the next generation, novel-designed drugs changing the DNA methylation status can reverse the detrimental effects of ELS in adulthood [109].

## 10 COVID-19-RELATED STRESS AND NEUROPSYCHIATRIC SEQUELAE

Although SARS-CoV-2 was initially described as a single-stranded RNA virus affecting the respiratory system and leading to severe cases of pneumonia, accumulating evidence suggests that coronavirus disease 2019 (COVID-19) may include symptoms and signs from other systems, including the central nervous system (CNS). Indeed, COVID-19 was shown to invade and lead to damage of the CNS *via* hosting by both neurons and immune cells, ultimately leading to neuropsychiatric complications [110]. Several studies reported patients with cognitive impairment and memory loss during the acute phase of infection. Furthermore, the implementation of restrictive measures during local lockdowns caused physical and social isolation, which was a severe and long-acting stressor for pregnant women, children and adolescents [110-114]. Undoubtedly, all these factors precipitated PTSD, depression, anxiety disorders, and psychosis, especially in adolescents in whom emotional regulation is tightly regulated [115, 116].

## CONCLUSION AND FUTURE PERSPECTIVES

The majority of scientific data supports a strong impact of early-life stress on child development and behavior. Indeed, exposure to early-life stressors has been associated with an increased risk for ADHD and ASD in the offspring. However, further and larger polycentric studies are undoubtedly needed since there are still some inconsistencies in the scientific literature [57]. The evolving biomedical field of epigenetics has shed light on the mechanisms through which stressors during critical periods of life, such as prenatal life, childhood and adolescence, may leave deep imprints on the human expressed genome, increasing the risk for depression, suicide attempts, metabolic syndrome, substance abuse, neurodevelopmental disorders, and many other adverse consequences. DNA methylation and ncRNAs seem to play a fundamental role in epigenetic modifications linking ELS to neurodevelopment and behavior. Importantly, these mechanisms are currently under intense investigation in order to identify new pharmacological targets with beneficial therapeutic outcomes and fewer adverse effects. To this end, the study of behavioral epigenetics is evolving at the epicenter of stress research, significantly contributing to our better understanding of the impact of early life stress on child neurodevelopment and behavior, as well as on the quality of adult life. As far as management is concerned, we recommend the implication of stress management methods, including progressive muscle relaxation, diaphragmatic breathing, guided imagery and cognitive restructuring, in order to alleviate the clinical manifestations of the above-discussed chronic diseases. Undoubtedly, future and large studies are needed to prove and support the effectiveness of such methods in reducing inappropriate stress responses. However, a growing body of evidence paves the way for future development [112, 113].

## LIST OF ABBREVIATIONS

ACEs: Adverse Childhood Experiences
ASD: Autism Spectrum Disorder
ATP: Adenosine Triphosphate
BZD: Benzodiazepines
CNS: Central Nervous System
CRH: Corticotropin-releasing Hormone
GH: Growth Hormone
GR: Glucocorticoid Receptor
GREs: Glucocorticoid Response Elements
hGR: Human Glucocorticoid Receptor
HPA: Hypothalamic-pituitary-Adrenal
HPT: Hypothalamic-pituitary-thyroid
LC: Locus Coeruleus
MAPK: Mitogen-activated Protein Kinase
MR: Mineralocorticoid Receptor
NP: Neuropeptide
PVN: Paraventricular Nuclei
